# Right ventricular preload and afterload challenge induces contractile dysfunction and arrhythmia in isolated hearts of dystrophin‐deficient male mice

**DOI:** 10.14814/phy2.16004

**Published:** 2024-04-24

**Authors:** Andrew Behrmann, Jessica Cayton, Matthew R. Hayden, Michelle D. Lambert, Zahra Nourian, Keith Nyanyo, Brooke Godbee, Laurin M. Hanft, Maike Krenz, Kerry S. McDonald, Timothy L. Domeier

**Affiliations:** ^1^ Medical Pharmacology and Physiology University of Missouri Columbia Missouri USA; ^2^ Department of Veterinary Pathobiology University of Missouri Columbia Missouri USA; ^3^ Dalton Cardiovascular Research Center University of Missouri Columbia Missouri USA

**Keywords:** cardiac arrhythmia, mdx mouse model, muscular dystrophy, pulmonary hypertension, right ventricle, ventricular tachycardia

## Abstract

Duchenne muscular dystrophy (DMD) is an X‐linked recessive myopathy due to mutations in the dystrophin gene. Diaphragmatic weakness in DMD causes hypoventilation and elevated afterload on the right ventricle (RV). Thus, RV dysfunction in DMD develops early in disease progression. Herein, we deliver a 30‐min sustained RV preload/afterload challenge to isolated hearts of wild‐type (Wt) and dystrophic (Dmd^mdx‐4Cv^) mice at both young (2–6 month) and middle‐age (8–12 month) to test the hypothesis that the dystrophic RV is susceptible to dysfunction with elevated load. Young dystrophic hearts exhibited greater pressure development than wild type under baseline (Langendorff) conditions, but following RV challenge exhibited similar contractile function as wild type. Following the RV challenge, young dystrophic hearts had an increased incidence of premature ventricular contractions (PVCs) compared to wild type. Hearts of middle‐aged wild‐type and dystrophic mice had similar contractile function during baseline conditions. After RV challenge, hearts of middle‐aged dystrophic mice had severe RV dysfunction and arrhythmias, including ventricular tachycardia. Following the RV load challenge, dystrophic hearts had greater lactate dehydrogenase (LDH) release than wild‐type mice indicative of damage. Our data indicate age‐dependent changes in RV function with load in dystrophin deficiency, highlighting the need to avoid sustained RV load to forestall dysfunction and arrhythmia.

## 1 INTRODUCTION

Duchenne muscular dystrophy (DMD) is a progressive and degenerative myopathy that occurs due to mutations in the *DMD* gene on the X‐chromosome encoding the cytoskeletal protein dystrophin (Duan et al., 2021; Wicklund, 2013). Dystrophin is the main cytoplasmic component of the dystrophin‐glycoprotein complex that is present in all striated muscle fibers and plays a key role in maintaining the integrity of the sarcolemma by linking it to the actin cytoskeleton (Gao & McNally, 2015; Valera et al., 2021). Skeletal muscle lacking dystrophin is highly susceptible to contraction‐induced sarcolemmal damage and cellular necrosis (Vilquin et al., 1998; Weller et al., 1990). Cardiomyocytes lacking dystrophin have also shown sarcolemmal damage with passive distention, which led to a stiffened, hypercontractile state and eventual cell death due unregulated influx of calcium and fibrotic damage (Bremner et al., 2022; Yasuda et al., 2005).

Clinically, DMD is observed in males and is the most common muscular dystrophy, effecting 19.8 per 100,000 live male births and having a pooled global prevalence of 7.1 per 100,000 males (Crisafulli et al., 2020). Patients with DMD experience progressive skeletal muscle wasting and diaphragmatic weakness leading to severe respiratory complications. Eventually, DMD patients lose almost all ambulatory function and have a shortened life expectancy of 30–40 years with the main cause of death being cardiopulmonary failure (Mercuri et al., 2019). It has been well documented that respiratory function, pulmonary vascular resistance (RV afterload), and RV function are tightly coupled and that elevated pulmonary vascular resistance can lead to deleterious effects on the RV (Bernardo et al., 2020; Simmons et al., 1961). In both patients and in animal models, the diaphragm weakness in DMD leads to pulmonary hypertension, increased RV afterload, and RV failure (Barbin et al., 2016; Huang et al., 2011; Ishizaki et al., 2008; Yotsukura et al., 1988). Patients suffering from DMD are also at increased risk of suffering from ventricular arrhythmia (Yotsukura et al., 1999). It is crucial to gain a better understanding of both the mechanisms and triggers of RV dysfunction and ventricular arrhythmia in dystrophic patients to improve outcomes.

The dystrophin‐deficient male mdx mouse displays early‐onset fibrosis within the diaphragm and RV, making it a clinically relevant model to study the intricate coupling of cardiopulmonary function (Barbin et al., 2016; McGreevy et al., 2015; Mele et al., 2021). For instance, genetic expression of dystrophin or utrophin in the diaphragm can rescue cardiac function in dystrophic mouse models (Crisp et al., 2011; Tinsley et al., 1998), which highlights the key role of respiratory function and pulmonary vascular resistance in DMD cardiac disease progression. Notably, RV dysfunction has been shown to precede left ventricular dysfunction in dystrophic mice starting at 3 months of age, showing an increased end systolic volume and reduced RV ejection fraction (Stuckey et al., 2012). RV fibrosis has been shown to precede fibrosis of the left ventricle and interventricular septum in dystrophic mice, yet increasing RV preload via abdominal compression did not reveal significant differences between dystrophic and wild‐type control hearts (Meyers & Townsend, 2015). This necessitates additional investigations into RV function not only in a dystrophic mouse model but for other rodent models to examine the complex interplay of the cardiopulmonary system.

Hence, the purpose of the current study is to test the hypothesis that the dystrophic mouse RV is susceptible to dysfunction and damage in response to a sustained elevation in RV load using a RV‐specific working heart model. We test this hypothesis in both young and middle‐aged wild‐type and dystrophic mice to provide additional insight on RV function with aging in DMD.

## 2 METHODS

### 2.1 Animal model

All protocols in this paper involving animals were performed in accordance with the Animal Care and Use Committee (Approval reference number 35701) of the University of Missouri, and conform with US regulations involving animal experiments. This study used male wild‐type C57Bl/6 mice (Wt) and male dystrophin‐deficient *Dmd* ^*mdx‐4Cv*^ mice (Jackson Labs strain #002378) that were 2–6 months (young) or 8–12 months (middle‐aged) of age.

### 2.2 Solutions

A modified Krebs–Henseleit buffer (KHB) was used for working heart perfusion. The KHB contained the following (in mmol/L): 117 NaCl, 4.7 KCl, 1.2 MgSO4, 1.2 KH2PO4, 25 NaHCO3, 11.1 glucose, 0.4 caprylic acid, 1 pyruvate, 0.0023 Na EDTA, and 1.8 CaCl2. Solution was warmed to 37°C in water‐jacketed glassware and maintained via a recirculating pump (Radnoti, #170051G).

### 2.3 Isolated working heart

**FIGURE 1:**
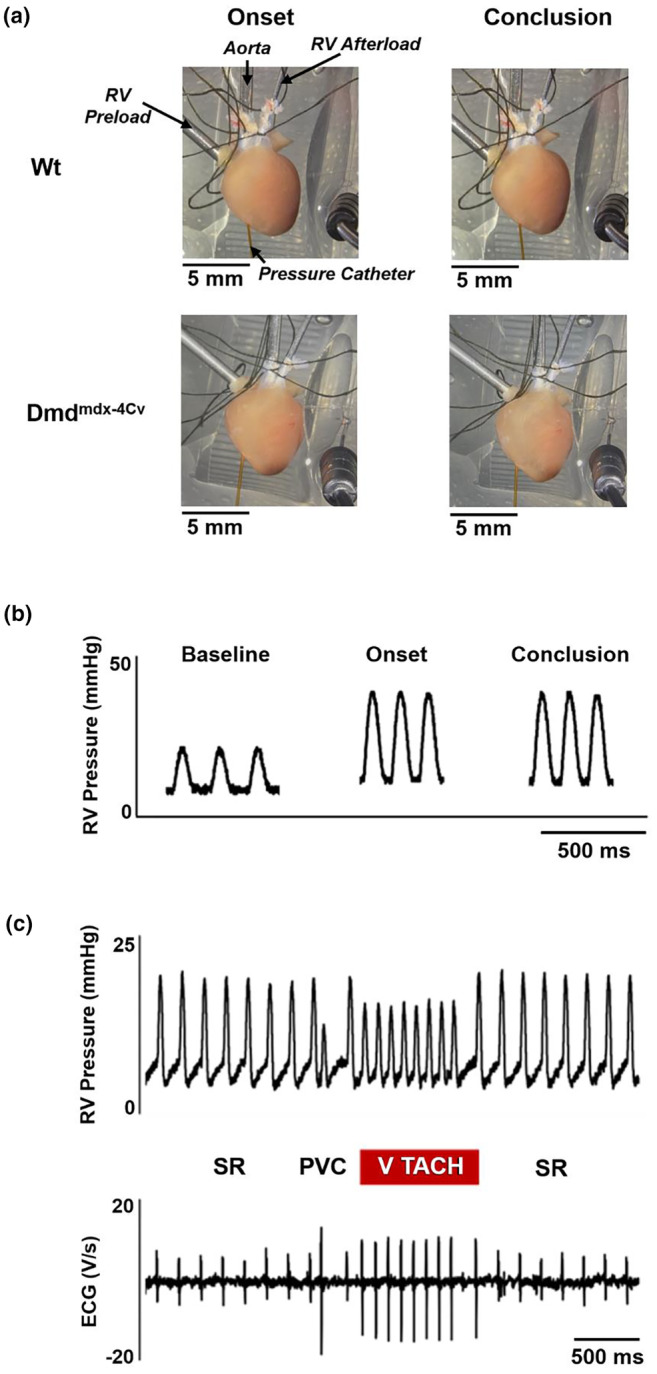
Experimental preparation and example recordings. (a) Isolated heart preparation of Wt (upper images) and Dmd^mdx‐4Cv^ (lower images) heart at the onset (left panels) and conclusion (right panels) of RV preload/afterload challenge. Aortic cannula (*Aorta*), pulmonary artery cannula (*RV Afterload*), right atrial cannula (*RV Preload*), and RV pressure catheter (*Pressure Catheter*) are labeled in first image for reference. Black ECG lead shown in bottom right of each image. (b) Example RV pressure recordings of Wt heart at baseline, at the onset of preload/afterload challenge, and at the conclusion of the preload/afterload challenge. (c) RV pressure traces (upper) and ECG traces (lower) of middle‐aged Dmd^mdx‐4Cv^ heart exhibiting sinus rhythm (SR), a premature ventricular contraction (PVC), and short bout of ventricular tachycardia (V TACH).

Mice were injected with intraperitoneal ketamine (NDC: 11695–0703‐1):xylazine (NDC: 61133‐6017‐1) (100 mg/kg:5 mg/kg), and once there was an absence of the pedal withdrawal reflex, hearts were rapidly excised within 30 s (Veteto et al., 2020). Once excised, hearts were immediately placed in a cold (4°C) KHB buffer. The hearts were cleared of excess lung, adipose, and aortic tissue, then submerged in a cold KHB bath also lacking calcium, and then cannulated via the aorta (60 mmHg afterload) to deliver warm (37°C) oxygenated KHB with 1.8 mM calcium to perfuse the coronary circulation (Langendorff mode) (Nyman et al., 2024). Any blood and cold KHB within the bath from the initial cannulation were suctioned out of the bath and replaced with warm oxygenated KHB containing 1.8 mM calcium before placing additional cannulas. The right atrium and the pulmonary artery were subsequently cannulated. Perfusate flow into the right atrium was closed via a stopcock during baseline conditions and opened to a preload of 10 mmHg for preload challenge. The pulmonary artery cannula was connected to a fluid column with a three‐way stopcock set level to the heart that was either open to atmosphere (baseline conditions) or connected to a 20 mmHg afterload column for the afterload challenge. A 25‐gauge needle was used to make a small insertion hole in the apical portion of the RV, and a 1.0 F Millar pressure catheter was inserted to monitor RV pressures using an FE231 Bio Amp and LabChart/Power 8.1 software (AD Instruments) (Figure 1a). The sampling rate was set to 1000 Hz with a 10 Hz low‐pass filter applied to improve signal‐to‐noise of pressure waveforms. After a 15‐min baseline period in Langendorff mode, the RV was then challenged with 10 mmHg preload and 20 mmHg afterload for 30 min. Based on previous investigations in our laboratory, under Langendorff mode conditions, left‐ventricular end‐diastolic pressures were 7 ± 3 mmHg in Wt and 5 ± 2 mmHg in Dmd^mdx‐4Cv^ (unpublished data), while left‐ventricular systolic pressures were 71 ± 17 in Wt and 68 ± 14 in Dmd^mdx‐4Cv^ (Haffner et al., 2023). For the current study, RV end‐diastolic pressures during Langendorff mode were 3 ± 0.6 in young Wt and 3 ± 0.4 in young Dmd^mdx‐4Cv^, and 3 ± 0.3 in middle‐aged Wt and 3 ± 0.3 in middle‐aged Dmd^mdx‐4Cv^. Right ventricular pressure development (P_dev_) and arrhythmias were assessed over 3–5‐min intervals: (1) The last 5 min of baseline Langendorff mode conditions; (2) initial 5 min of preload/afterload challenge (maximum response to pressure elevation); and (3) the last 5 min of 30 min post‐RV challenge (Figure 1b). All measurements were gathered as an average of 5–10 s of steady‐state pressure and ECG tracings. Peak RV pressures exceeded pressure of the afterload column, and thus fluid was ejected from the RV out of the RV afterload column. An inclusion criterion was established that hearts needed to develop 20 mmHg at the onset of the load challenge to eject perfusate and avoid stagnant RV flow (Lou et al., 2011). Three hearts in the study did not meet this inclusion criteria at the onset of the protocol (1 young Wt, 1 young Dmd^mdx‐4Cv^, and 1 middle‐aged Dmd^mdx‐4Cv^); therefore, they were excluded from this study.

### 2.4 Electrocardiogram (ECG) measurements

Arrhythmias were assessed at baseline and during the RV load challenge using a set of three 1.5 mm shrouded socket monopolar 29‐gauge MLA1213 needle electrodes connected to a FE231 Bio Amp and LabChart/Power 8.1 (AD Instruments). Electrode position was adjusted as needed to gain clear distinction of each cardiac cycle's P wave and QRS complex. A premature ventricular contraction (PVC) was defined as a premature ventricular complex on the ECG waveform that either preceded or was completely independent of an atrial‐driven P wave. The QRS complexes of PVCs tended to be of higher amplitude and longer duration compared to the QRS complexes of sinus rhythm, allowing for clear identification. The incidence of ventricular arrhythmias within each minute of the 5 ‐min intervals was quantified using a 0‐1‐2 scoring system. A score of 0 indicating no arrhythmias within that minute, a score of 1 indicating isolated PVCs, and score of 2 indicating the presence of salvos of 3 or more PVCs or runs of ventricular tachycardia/fibrillation (Figure 1c) (Haffner et al., 2023). The 5‐min average arrhythmia score for each heart was recorded for further data analysis and comparison.

### 2.5 LDH effluent collection

Following the RV load challenge, coronary effluent was collected for detection and quantification of lactate dehydrogenase (LDH) as an index of cardiac damage, which was visually observed in the right ventricle of the Dmd^mdx‐4Cv^ hearts (Figure 1a). Lactate dehydrogenase was quantified using the Promega luminescent LDH‐Glo 105TM Cytotoxicity Assay kit following manufacturer protocol (Promega, Madison, WI, USA; catalog number: J2381).

### 2.6 Statistical analysis

All samples are reported as mean ± standard deviation. To compare group means, a two‐sided student's t‐test was performed in Graph Pad/Prism. Analysis of LDH bioluminescent assay was performed using a one‐tailed Mann–Whitney *U*‐Test. A *p*‐value <0.05 was considered statistically significant. ECG data were analyzed by both an experimentalist who was unblinded to the genotype and confirmed by analysis performed by individuals who were blinded to the genotype of the animals to increase rigor of the data analysis (Percie du Sert et al., 2020).

## 3 RESULTS

### 3.1 Young

**FIGURE 2:**
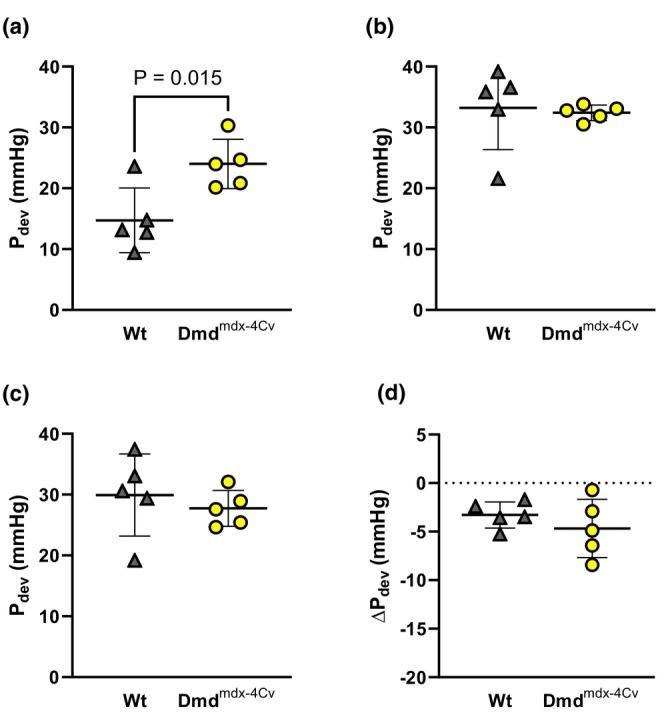
Right Ventricular Pressure Development of Young wild‐type and dystrophic Dmd^mdx‐4Cv^ hearts. Summary data of Developed Pressure (P_dev_) of Young Wt (*n* = 5, gray triangles) and Dmd^mdx‐4Cv^ (*n* = 5, yellow circles) at last 5 min of baseline (a), first 5 min of preload/afterload challenge (b), and last 5 min of preload/afterload challenge (c). Change in P_dev_ from the first 5 min to the last 5 min of the preload/afterload challenge shown in (d), Independent‐samples t‐test.

**FIGURE 3:**
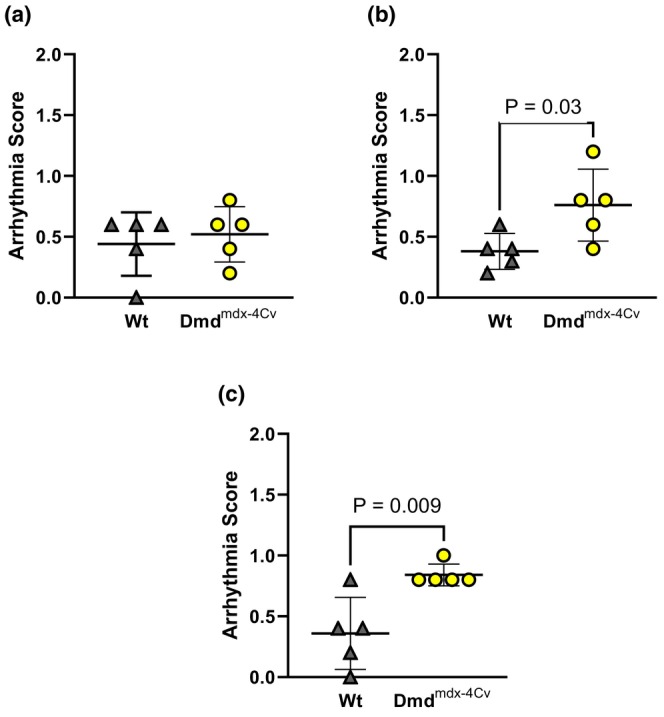
Ventricular Arrhythmia incidence in Young wild‐type and dystrophic Dmd^mdx‐4Cv^ hearts. Summary data of average arrhythmia scores of Young Wt (*n* = 5, gray triangles) and Dmd^mdx‐4Cv^ (*n* = 5, yellow circles) at last 5 min of baseline (a), first 5 min of preload/afterload challenge (b), and last 5 min of preload/afterload challenge (c), Independent‐samples *t*‐test.

**FIGURE 4:**
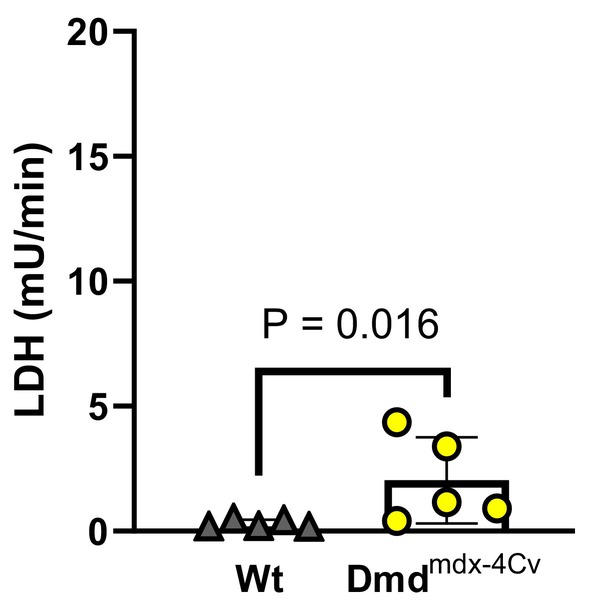
Cardiac lactate dehydrogenase release following preload/afterload challenge in Young wild‐type and dystrophic Dmd^mdx‐4Cv^ hearts. Summary data of lactate dehydrogenase (LDH) release of Young Wt (*n* = 5, gray triangles) and Dmd^mdx‐4Cv^ (*n* = 5, yellow circles) hearts at the conclusion of the preload/afterload challenge, one‐tailed Mann–Whitney *U*‐test.

Compared to Wt mice under baseline conditions, young Dmd^mdx‐4Cv^ hearts exhibited greater RV P_dev_ (Figure 2a). Both young groups exhibited similar P_dev_ at the onset (Figure 2b) and conclusion (Figure 2c) of the combined preload/afterload challenge. Change in pressure development from the onset to the conclusion of the preload/afterload challenge did not significantly differ between young Wt and young Dmd^mdx‐4Cv^ mice (Figure 2d). Average heart rates did not significantly differ between young Wt and Dmd^mdx‐4Cv^ hearts during baseline conditions (Heart rate: 368 ± 29 beats/min Wt vs. 355 ± 45 beats/min Dmd^mdx‐4Cv^, *p* = 0.56), initial loaded conditions (Heart rate: 433 ± 24 beats/min Wt vs. 447 ± 18 beats/min Dmd^mdx‐4Cv^, *p* = 0.14), and the conclusion of loaded conditions (Heart rate: 401 ± 29 beats/min Wt vs. 426 ± 22 beats/min Dmd^mdx‐4Cv^, *p* = 0.16). Arrhythmia incidence was low in both young Wt and young Dmd^mdx‐4Cv^ hearts during baseline conditions (Figure 3a), yet increased in Dmd^mdx‐4Cv^ hearts at both the onset (Figure 3b) and conclusion of the preload/afterload challenge (Figure 3c). There were no bouts of ventricular tachycardia observed in young Wt or Dmd^mdx‐4Cv^ mice, only isolated PVCs. LDH release following the preload/afterload challenge was significantly higher in young Dmd^mdx‐4Cv^ hearts compared to young Wt hearts (Figure 4).

### 3.2 Middle‐aged

**FIGURE 5:**
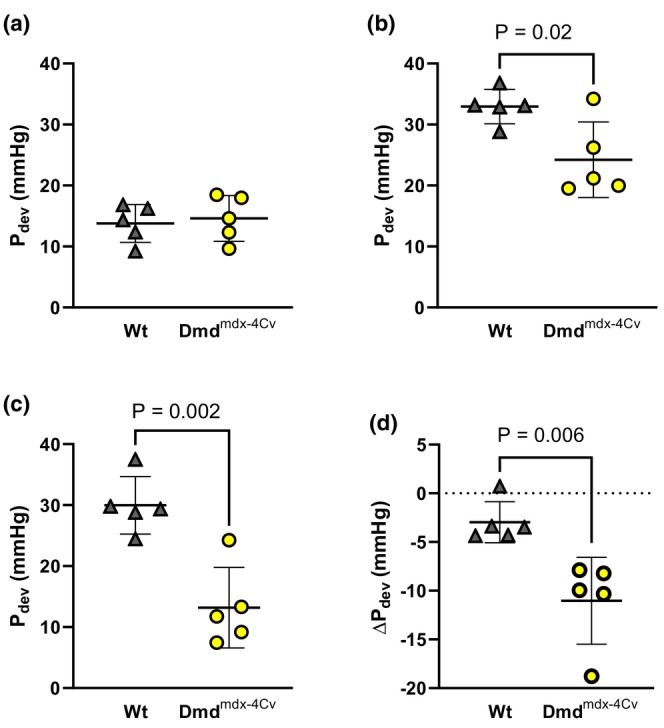
Right Ventricular Pressure Development of Middle‐aged wild‐type and dystrophic Dmd^mdx‐4Cv^ hearts. Summary data of Developed Pressure (P_dev_) of Middle‐aged Wt (*n* = 5, gray triangles) and Dmd^mdx‐4Cv^ (*n* = 5, yellow circles) at last 5 min of baseline (a), first 5 min of preload/afterload challenge (b), and last 5 min of preload/afterload challenge (c). Change in P_dev_ from the first 5 min to the last 5 min of the preload/afterload challenge shown in (d), Independent‐samples *t*‐test.

**FIGURE 6:**
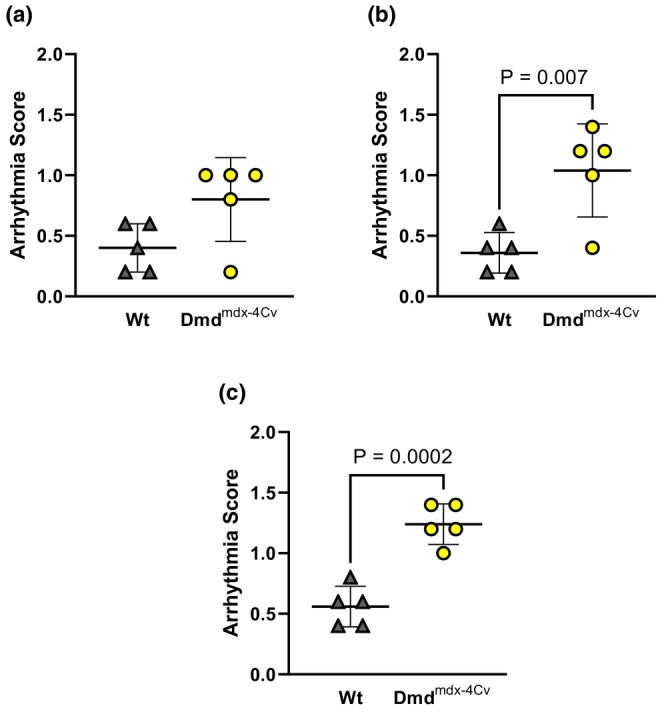
Ventricular Arrhythmia incidence in Middle‐aged wild‐type and dystrophic Dmd^mdx‐4Cv^ hearts. Summary data of average arrhythmia scores of Middle‐aged Wt (*n* = 5, gray triangles) and Dmd^mdx‐4Cv^ (*n* = 5, yellow circles) at last 5 min of baseline (a), first 5 min of preload/afterload challenge (b), and last 5 min of preload/afterload challenge (c), Independent‐samples t‐test.

**FIGURE 7:**
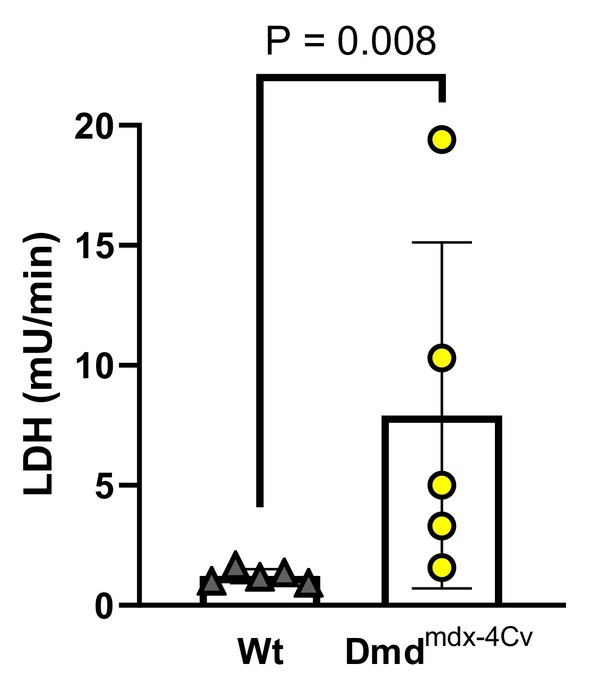
Cardiac lactate dehydrogenase release following preload/afterload challenge in Middle‐aged wild‐type and dystrophic Dmd^mdx‐4Cv^ hearts. Summary data of lactate dehydrogenase (LDH) release of Middle‐aged Wt (*n* = 5, gray triangles) and Dmd^mdx‐4Cv^ (*n* = 5, yellow circles) hearts at the conclusion of the preload/afterload challenge, one‐tailed Mann–Whitney *U*‐test.

In middle‐aged mice, hearts of both Wt and Dmd^mdx‐4Cv^ groups exhibited similar P_dev_ under baseline conditions (Figure 5a). However, P_dev_ was lower in Dmd^mdx‐4Cv^ hearts at both the onset (Figure 5b) and conclusion (Figure 5c) of the preload/afterload challenge. Furthermore, during the sustained load challenge, middle‐aged Dmd^mdx‐4Cv^ hearts exhibited a significant loss of RV contractile function (Figure 5d). Average heart rate did not significantly differ between middle‐aged Wt and Dmd^mdx‐4Cv^ hearts during baseline conditions (Heart rate: 386 ± 25 beats/min Wt vs. 384 ± 19 beats/min Dmd^mdx‐4Cv^, *p* = 0.89), initial loaded conditions (Heart rate: 410 ± 15 beats/min Wt vs. 409 ± 18 beats/min Dmd^mdx‐4Cv^, *p* = 0.19), and the conclusion of loaded conditions (Heart rate: 423 ± 18 beats/min Wt vs. 425 ± 24 beats/min Dmd^mdx‐4Cv^, *p* = 0.88). Arrhythmia incidence was similar between middle‐aged Wt and middle‐aged Dmd^mdx‐4Cv^ hearts during baseline conditions (Figure 6a). However, at the onset (Figure 6b) and conclusion (Figure 6c) of the combined preload/afterload challenge, middle‐aged Dmd^mdx‐4Cv^ hearts exhibited significantly higher arrhythmia scores. Bouts of ventricular tachycardia were only observed in middle‐aged Dmd^mdx‐4Cv^ hearts (*n* = 4/5 hearts). Middle‐aged Dmd^mdx‐4Cv^ hearts also had significantly greater LDH release compared to middle‐aged Wt hearts at the conclusion of the preload/afterload protocol (Figure 7).

## 4 DISCUSSION

This study examined RV function in a Dmd^mdx‐4Cv^ mouse model using an isolated working heart method aimed at fundamentally mimicking the RV disease phenotype previously described in dystrophic mice and patients (Barbin et al., 2016; Meyers & Townsend, 2015; Yotsukura et al., 1988). In vivo MRI of mdx mice noted that RV dysfunction is seen as early as 3 months, showing elevated end systolic volume and a decreased ejection fraction (Stuckey et al., 2012). Notably, our study found that during baseline conditions, young Dmd^mdx‐4Cv^ hearts were hypercontractile compared to Wt, but middle‐aged Wt and Dmd^mdx‐4Cv^ hearts displayed similar contractile function. This discrepancy may be explained by the need for the Dmd^mdx‐4Cv^ heart to be in a hypercontractile state to compensate for the elevated afterload seen in vivo (Li et al., 2014). However, our data suggest that dystrophic hearts lose an adaptive hypercontractile mechanism with aging, and exhibit a diminished contractile response to increased ventricular load, which worsens with age (Figure 5b). Furthermore, the middle‐aged Dmd^mdx‐4Cv^ hearts exhibited a remarkable loss in RV contractile function with sustained elevation in RV load (Figure 5c,d). The elevated RV afterload seen in DMD‐induced pulmonary hypertension can result in cardiac afterload stress and damage (Haffner et al., 2023; Seo et al., 2014; Yasuda et al., 2005), which can lead to cellular hypercontractility and eventual cell death. Our study found similar results with Dmd^mdx‐4Cv^ hearts releasing significantly more LDH compared to Wt hearts of the same age, regardless of maintained (young) or diminished (middle‐age) cardiac function throughout the load challenge. Several biomarkers can be used to examine cardiac damage in the setting of muscular dystrophy, including LDH (Barnabei & Metzger, 2012; Burelle et al., 2010; Haffner et al., 2023), creatine kinase (Chang et al., 2016; Jearawiriyapaisarn et al., 2010; Law et al., 2018), and Troponin I (De Giorgio et al., 2023; Lopez et al., 2017; Matsumura et al., 2007; Uryash et al., 2021). Indeed, there are advantages and disadvantages of each including size (Creatine Kinase preferred) and specificity (Troponin I preferred). In the isolated organ environment, a general marker such as LDH is also sufficient to assess muscle damage, particularly given the nature of the challenge and stretch‐induced damage commonly observed in dystrophic cardiac muscle (Barnabei & Metzger, 2012; Haffner et al., 2023; Yasuda et al., 2005). Presumably, disease progression in vivo will result in the damaged tissue being replaced by fibrous tissue, which, in turn, further worsens overall RV function as dystrophic mice continue to age.

The dystrophic mouse models have previously shown susceptibility to ventricular arrhythmias while using programmed electrical stimulation and a catecholamine challenge (Ather et al., 2013; Wang et al., 2018). Similarly, in vivo ECG recordings revealed an increased incidence of PVCs in dystrophic versus wild‐type mice, which could trigger persistent ventricular tachycardias (Fauconnier et al., 2010). Indeed, our group recently determined that increased preload in the left ventricle of Dmd^mdx‐4Cv^ hearts led to calcium handling abnormalities and increased incidence of PVCs (Haffner et al., 2023). Given the sensitivity of dystrophic myocardium to stretch‐induced calcium overload and damage, it is likely that the LDH release and arrhythmic beats originated from whichever ventricle experiences the greater load and thus greater extent of stretch. Thus, the finding of greater differences in LDH release and arrhythmia scores following increased RV load as well as the visually apparent damage in the RV after stretch (Figure 1a) suggests an RV origin of cardiac pathology. However, our current measurements cannot exclude a LV/septal origin of cardiac dysfunction due to deformation of the septal wall in response to elevated RV pressures.

The Langendorff heart model is a well‐established preparation to investigate cardiac function ex vivo and associates with several experimental advantages as well as disadvantages (Liao et al., 2012; Nyman et al., 2024). Advantages include the ability to study the heart in the absence of systemic factors allowing one to study biology inherent to cardiac muscle. For the present investigation, it is key to study RV stretch without concomitant homeostatic changes that may be observed in vivo. Isolated hearts also allow for the assessment of damage markers of the organ versus from other tissues (e.g., dystrophic skeletal muscle). With absence of murine neurohormonal factors in vivo, heart rate slows in the ex vivo environment, which facilitates diastolic filling time and may therefore provide a better approach to investigate the effects of chamber and cardiomyocyte stretch with increased load. As overdrive suppression of arrhythmias occurs at high heart rates, the slower heart rate associated with ex vivo conditions may also facilitate measurements of spontaneous arrhythmogenic ventricular activity. Disadvantages of this model include that during baseline conditions (i.e., no preload/afterload supplied), the ventricles still receive some load from venous return of effluent through the Thebesian circulation or the coronary sinus (Ciszek et al., 2007). In addition, the LV was not supplied with defined preload during the RV load challenge, which may lead to atypical septal movement. However, such atypical wall movement has been noted in mdx mice and is a commonly observed phenomenon in clinical scenarios of pulmonary hypertension and excessive RV pressures (Palau‐Caballero et al., 2017; Stuckey et al., 2012). Changes in coronary vasoreactivity have been reported in dystrophic mouse hearts, as have impairments in cardiac energetics (Mitchell et al., 2021; Stelter et al., 2016; Stevens et al., 2024). Thus, while the 60‐mmHg coronary perfusion pressure is common in Langendorff heart preparations, the lower pressure and decreased oxygen‐carrying capacity of the saline‐perfused Langendorff preparation may unmask organ‐level deficits in myocardial perfusion or energetics in the setting of muscular dystrophy.

In conclusion, our study suggests that baseline RV hypercontractility displayed in young dystrophic hearts may be a temporary, adaptive mechanism to compensate for the pulmonary hypertension observed in dystrophic mice (Barbin et al., 2016; Meyers & Townsend, 2015; Zhang et al., 2008). As baseline hypercontractility dissipates in middle‐aged dystrophic hearts, the ability to withstand prolonged elevated loading conditions also declines. An inability to meet afterload demands in vivo may lead to increased end systolic volumes and thus, further elevation in preload (Stuckey et al., 2012; Zhang et al., 2008). There is extensive data that indicates the RV of dystrophic mice displays severe fibrosis that worsens with age (De Giorgio et al., 2023; Li et al., 2009, 2014; Meyers & Townsend, 2015; Stuckey et al., 2012). This may serve as the substrate for ventricular arrhythmia upon triggers induced by stretch‐induced calcium mishandling, thus increasing arrhythmia incidence and severity in middle‐aged dystrophic hearts compared to wild‐type mice of similar age (compare Figure 3 and Figure 6) (Centurión et al., 2019; Haffner et al., 2023; Van Erp et al., 2010). Clinically, these data highlight that DMD patients should avoid prolonged situations of excessive RV load (e.g., suboptimal ventilatory support) due to risk the of fatal ventricular arrhythmia.

## FUNDING INFORMATION

This work was supported by the National Heart Lung and Blood Institute at the National Institutes of Health (NIH) R01HL136292 and a University of Missouri System Tier 2 Award – Research and Creative Works Strategic Investment Program (Krenz, McDonald, Co‐PIs).
